# A Literary (Medical) Curiosity

**Published:** 1836-07

**Authors:** 


### A LITERARY (MEDICAL) CURIOSITY.

In the Danish Medical Journal, *Bibliothek for Læger*, No. I. for 1836, Professor O. Bang, of Copenhagen, has published an *Essay on the Pathology of Diseases*, in the form of a Poem in rhyme! It consists of forty-three octave stanzas, and is entitled "*Livets Kamp med Døden eller Sygdommens Oprindelse.*" We are informed by those who are better critics than we can pretend to be in such matters, that the poem is really a valuable one, and that the verses are both harmonious and graceful, even where they have to treat of the most trivial circumstances. The medical principles inculcated in the poem are purely Hippocratic, the author deriving all diseases from the efforts of nature to remove what would injure or destroy the body. If Dr. Bang had not preferred being an excellent physician, he certainly might have become a famous poet.

